# The EBV-MS connection: the enigma remains

**DOI:** 10.3389/fimmu.2024.1466339

**Published:** 2024-08-29

**Authors:** A. van de Waterweg Berends, B. Broux, B. Machiels, L. Gillet, N. Hellings

**Affiliations:** ^1^ Neuro-Immune Connections and Repair Lab, Biomedical Research Institute, Department of Immunology and Infection, UHasselt, Diepenbeek, Belgium; ^2^ University MS Center, Campus Diepenbeek, Diepenbeek, Belgium; ^3^ Laboratory of Immunology and Vaccinology, Faculty of Veterinary Medicine, Fundamental and Applied Research for Animals and Health (FARAH), ULiège, Liège, Belgium

**Keywords:** autoimmunity, multiple sclerosis, herpesviruses, Epstein-Barr virus, molecular mimicry 1

## 1 Introduction

In multiple sclerosis (MS) patients, the central nervous system (CNS) is affected by the infiltration of inflammatory immune cells, resulting in demyelination and axonal loss. Although the exact trigger for the activation of autoimmune responses against the CNS has not been elucidated yet, a genetic predisposition in combination with environmental factors seems essential to develop MS. Therefore, MS is considered to be a multifactorial disease. The main environmental factors increasing the risk of MS are obesity, smoking, low vitamin D levels, and Epstein-Barr virus (EBV) infection (1). EBV is a human gammaherpesvirus and its life cycle starts after infection of epithelial cells where it replicates and then spreads to B cells to establish lifelong latency. Latent EBV can be reactivated in memory B cells. EBV was first linked to MS almost 40 years ago. The first hypothesis was developed after identifying many similarities between the occurrence and latitude gradient of MS and infectious mononucleosis (IM) caused by EBV, and therefore MS was proposed to be a complication of an EBV infection (2). EBV is also reported to be associated with MS relapses (3), however, here we emphasize the involvement in MS etiology rather than in disease progression and we highlight the progress made in the field and provide suggestions for future research.

## 2 Epidemiological evidence

A lot of epidemiological evidence is available that shows the association between EBV and MS. For example, anti-EBV antibodies are elevated 5 or more years before the onset of MS symptoms, and the most consistent observations are found for antibodies against EBV nuclear antigens (e.g. EBNA-1 and EBNA-2) which are expressed during a latent infection (4). Besides antibodies against the latent-expressed EBNA, also antibodies against the lytic-expressed viral capsid antigen (VCA) are elevated in MS patients (5) and correlates with disease activity (6). Also, the risk of MS can be neglected in individuals who are seronegative for EBV (7). In immunocompetent individuals, an EBV infection is often mild and self-limiting (8), especially when infected during childhood. However, when an individual is exposed to EBV during adolescence, the chances of developing IM after an EBV infection are increased (9). IM-affected individuals have a 2-3 times higher MS risk than individuals who are infected with EBV during childhood and did not develop IM (10, 11).

In most high MS risk areas, the hygiene standards are higher and people are generally exposed to EBV later in life while in low MS risk areas, there is a high chance of an early EBV infection during childhood (12, 13). Therefore, the epidemiological observation that there is a reduction in MS risk when migrating from a high to a low MS risk area could only be explained by EBV if migration occurred in early childhood resulting in earlier exposure to EBV and a lower risk for developing IM. While many studies show that the effects after migration are age independent (14, 15), Martyn et al. showed that this reduction in MS risk is documented for migration at all ages (16). This observation seems in conflict with the EBV-MS hypothesis and suggests that additional factors such as EBV strain differences or differences in exposure to other important synergistic exposure are contributing to overall MS risk.

Recently, Bjornevik et al. published the largest and most comprehensive study on the relationship between EBV and MS that has been conducted so far. This elegantly performed study involved a 20-year follow-up of over 10 million US army personnel from which serum samples were collected during this time (17). Of these people, 955 individuals were diagnosed with MS. Serum samples that were insufficient to perform EBV serology were excluded which in the end led to 801 MS cases and 1566 matched controls. A higher rate of prior EBV seroconversion was found in individuals who eventually developed MS compared to those who remained healthy. Moreover, a 32-fold increased risk for MS was seen in people who seroconverted compared to those who remained EBV-negative and a good correlation with neurofilament light (NFL) serum levels, a marker for neurodegeneration was seen. Based on this, the researchers suggest that EBV is the leading cause of MS.

This study got significant attention, reigniting interest in this topic, and has been cited over 400 times. While this study is important and statistically rigorous, certain caution should still be taken regarding the conclusions related to actual causality in this paper. More specifically, 1) An impressive number of 10 million individuals and 955 MS cases were monitored, but only 32 MS cases seroconverted prior to disease development. While statistics were convincing, readers should realize that the conclusions on actual causality and the positive correlation with NFL levels are based on this relatively small sample size. 2) One MS case remained EBV negative. This number seems neglectable, however, based on the sample size (n=33) this is still 3%. Although this may not contradict the possibility of causality, it refutes that EBV is necessary for the development of MS. 3) Any data regarding IM status is missing while it is reported that IM increases MS risk (18, 19). If the percentage of persons who developed IM after EBV infection is different between cases versus controls, this can significantly impact the hazard ratio reported here, especially since the cohort consisted of individuals who seroconverted later in life and thus have a higher risk for developing IM (10).

Drawing definitive conclusions on causality based on epidemiological studies remains challenging, primarily due to the widespread prevalence of EBV within the adult population. While this longitudinal study has made substantial efforts in this regard, caution is warranted when news items and editorials are stating that EBV is necessary for the development of MS. Even more so, when zooming in on pediatric MS. Two reports indicate that no less than 13-14% of pediatric MS patients compared to 26% of age and region-matched healthy controls are tested negative for EBV (20, 21). While these findings reaffirm the association between EBV and MS, they challenge the theory that EBV infection is necessary for MS development.

## 3 Mechanistic evidence

Several mechanisms have been proposed about how EBV could contribute to the immunopathology of MS, including molecular mimicry, bystander activation of autoimmune T cells, B cell transformation, and CNS tropism [for further readings see (22, 23)]. One of the best-documented mechanisms is the involvement of EBV-infected B cells in pathogenic mechanisms related to MS. However, until now the exact mechanistic explanation of how EBV infection contributes to MS remains speculative.

Lanz et al. recently provided further evidence for the molecular mimicry hypothesis. This is a phenomenon in which the virus shares structural similarities with host proteins and thus could lead to cross-activation of autoimmune reactive lymphocytes. The study demonstrated molecular mimicry between the EBV nuclear antigen EBNA1 and the glial cell adhesion protein that is expressed in the CNS by both astrocytes and oligodendrocytes (GlialCAM). They identified that one-third of the clonally expanded antibodies in the CSF of MS patients recognize EBNA-1 and part of them cross-reacted with GlialCAM (22). Although interesting, cross-reactivity between EBNA1 and GlialCAM was only found in 20 to 25% of the MS patients. This highlights that MS is not solely driven by this specific cross-reactivity. Lastly, antibody reactivity to EBNA-1 and GlialCAM is also seen in healthy controls, indicating that molecular mimicry is not sufficient to break immune tolerance and trigger autoimmunity. Moreover, vaccination against EBNA-1 prior to disease induction aggravated clinical symptoms in EAE, an animal model of MS. Even though, EBNA1 vaccination is pathogenic in mice, the role of EBNA1 antibodies in humans remains poorly understood.

To find a plausible explanation for why only certain individuals with EBNA-1-GlialCAM cross-reactivity develop MS and others do not, Vietzen et al. investigated the potential underlying immunological mechanisms (24). They identified distinct cytotoxic NK cell responses that control GlialCAM specific lymphocytes. Those protective NK responses are less functional in MS patients compared to healthy controls with similarly high levels of cross-reactive EBNA-1 IgG. A notable aspect of this study is that it elaborates on the Nature paper of Lanz et al. with a potential mechanical explanation. However, the presence of GlialCAM antibodies in MS patients is not universal, thus this does not comprehensively elucidate the mechanism concerning the involvement of EBV in all MS patients.

EBV could also indirectly influence susceptibility to MS by interacting with other pathogens. For example, EBV is known to reactivate endogenous retroviruses (HERVs) (25) which is associated with both MS development and progression (26, 27). The reactivation of HERVs can decrease oligodendrocyte maturation and myelination through innate immune responses and it can lead to the production of nitric oxide and pro-inflammatory cytokines (28). On the other hand, human cytomegalovirus (CMV) can lead to infectious mononucleosis and thus manifest in a similar manner as EBV. However, studies show that CMV infection did not increase MS risk or may even be protective (17, 29). CMV infection is associated with lower EBNA1 levels (29, 30), indicating that CMV might modulate immune responses towards EBV and could in this way affect MS risk.

## 4 Challenges

Not all EBV infections cause disease directly, thus identifying the factor that causes the disruption in immunotolerance in EBV-infected MS patients is crucial. Studying the underlying mechanism of how EBV is involved in MS is laborious. Using animal models can be challenging since humans are the only natural hosts for EBV. The murine virus related to EBV, murid herpesvirus 4 (MuHV-4) can be used to overcome this problem and to study the effect of gammaherpesvirus infection in mouse models of MS such as experimental autoimmune encephalomyelitis (EAE) (31, 32). MuHV-4 shows important biological similarities to EBV; both infect B cells, have similar immune clearance of the acute infection and establish a life-long latency in the host (33). However, some genes that encode the immune controlling of latency differ between EBV and MuHV-4 as they uniquely adapted to their host (34). Human immune system (HIS) mice, in which human immune cells and their progenitors are engrafted into immunodeficient mice, may provide a solution to this problem, since these mice can be infected with EBV (35, 36). A disadvantage remains that only the leukocytes are sensitive and resemble the human situation.

On the other hand, unraveling the mechanisms in humans is also very demanding and difficult. Given the high prevalence of EBV in the general population and the relative rarity of MS, assembling a cohort of EBV-seroconverted individuals who will develop MS is exceptionally challenging. Since seroconversion precedes MS development 5 to 7 years on average (17) long-term follow-up is needed and sampling and storing blood samples of all individuals would cost a fortune. The cohort used in Bjornevik’s study involving US army personnel illustrates this as the study starts with a pool of 10 million and ends up with only 32 cases that seroconverted and developed MS. To gain insights into potential mechanisms, investigating immune cell differences from these individuals could offer valuable answers.

## 5 Future prospects

There is undoubtedly a connection between EBV and MS. To further acknowledge EBV as causal factor, future studies on plausible mechanisms are highly needed. The most convincing evidence would emerge from a human study in which EBV seronegative individuals are administered a yet-to-be-released preventive EBV vaccine and followed up long-term to see whether any of them develop MS. Many clinical studies have been conducted with the aim of developing an effective EBV vaccine, but none have reached the finish line. While some could reduce the risk of IM, none of them were able to prevent EBV infection (37). Currently, the company Moderna is developing an EBV-based mRNA vaccine mRNA-1189 (NCT05164094), encoding EBV envelope glycoproteins (gp350, gB, gH/gL and pg42) which mediate entry in the host cells and testing this in seronegative adults. The vaccine aims at preventing EBV-induced IM and might even stop EBV infection, but it is still too early to conclude whether this vaccine is effective, since it is still in phase 1. Another point of argument is to determine the best timing of vaccination. If the idea would be to prevent EBV infection, immunization of infants should be considered. Also, it is worth contemplating the potential evolutionary advantages of viruses as it is postulated by the “old friends” hypothesis. For instance, mice infected with MuHV-4 are resistant to infections with heterologous pathogens (38, 39). Additionally, MuHV-4 infection has beneficial capacities for its host by protecting against allergic asthma through imprinting of the monocyte compartment (40). Therefore, a thorough understanding of the mechanisms that drive either the beneficial or detrimental effects is needed and needs to be taken into consideration when aiming for EBV vaccination to prevent MS development.

While a plethora of underlying EBV mediated mechanisms has been proposed to contribute to MS, one area that is understudied is the influence of the innate immune system. While most research has primarily concentrated on the involvement of lymphocytes in the EBV-MS connection, it is well established that EBV affects myeloid cells (41, 42). These cells also play major roles in the MS pathogenesis. Monocytes and macrophages are particularly of importance in the early stages of MS and could thus contribute to the idea that EBV initiates MS through priming myeloid cells. Primary EBV infection is known to alter the host cytokine response, potentially compromising the blood-brain barrier (BBB) and therefore making the CNS more prone to immune cell invasion resulting in CNS damage. Recently, the effect of EBV on BBB permeability has been tested *in vitro*, but *in vivo* results are lacking (43). Apart from the question of how EBV contributes to the etiology of MS, a substantial number of studies demonstrated that it might also aggravate existing disease (31, 32).

## 6 Conclusion

Based on the multitude of studies performed, a clear connection between EBV and MS emerges. However, drawing definitive conclusions regarding causality remains challenging. EBV is highly prevalent in the adult population, and nearly everyone is exposed to EBV once in their lifetime. Moreover, many other risk factors are associated with MS that are independent of EBV. Before assuming that the entire MS puzzle is solved, there is still a substantial amount of unknowns left to discover. EBV is thought to contribute to MS development through various pathways, involving multiple mechanisms. Unravelling the precise mechanism through which EBV may contribute to the development of MS could aid in establishing causation since intervening in this mechanism would provide another strategy to find causative proof. Until the mechanisms are uncovered, this topic will continue to be a subject of debate and limit the investments in EBV targeted therapies against MS.
